# mHealth App to Facilitate Remote Care for Patients With COVID-19: Rapid Development of the DrCovid+ App

**DOI:** 10.2196/38555

**Published:** 2023-02-07

**Authors:** Jamaica Pei Ying Tan, Michelle W J Tan, Rachel Marie Towle, Joanne Sze Win Lee, Xiaofeng Lei, Yong Liu, Rick Siow Mong Goh, Franklin Tan Chee Ping, Teck Choon Tan, Daniel Shu Wei Ting, Chen Ee Lee, Lian Leng Low

**Affiliations:** 1 Population Health and Integrated Care Office Singapore General Hospital Singapore Singapore; 2 Family Medicine and Continuing Care Singapore General Hospital Singapore Singapore; 3 Nursing Singapore General Hospital Singapore Singapore; 4 Institute of High Performance Computing Agency for Science, Technology and Research Singapore Singapore; 5 Office for Service Transformation Singapore Health Services Singapore Singapore; 6 Department of Ophthalmology Singapore National Eye Centre Singapore Singapore; 7 Duke-NUS Medical School National University of Singapore Singapore Singapore; 8 Innovation and Transformation Singapore Health Services Singapore Singapore; 9 Outram Community Hospital SingHealth Community Hospitals Singapore Singapore; 10 Centre for Population Health Research and Implementation SingHealth Regional Health System Singapore Singapore

**Keywords:** mobile health, mHealth, rapid development, remote care, COVID-19, hospital-at-home, mobile app, app development, virtual care, Telegram service, clinical care, continuity of care, digital health

## Abstract

**Background:**

The 2019 novel COVID-19 has severely burdened the health care system through its rapid transmission. Mobile health (mHealth) is a viable solution to facilitate remote monitoring and continuity of care for patients with COVID-19 in a home environment. However, the conceptualization and development of mHealth apps are often time and labor-intensive and are laden with concerns relating to data security and privacy. Implementing mHealth apps is also a challenging feat as language-related barriers limit adoption, whereas its perceived lack of benefits affects sustained use. The rapid development of an mHealth app that is cost-effective, secure, and user-friendly will be a timely enabler.

**Objective:**

This project aimed to develop an mHealth app, *DrCovid+*, to facilitate remote monitoring and continuity of care for patients with COVID-19 by using the rapid development approach. It also aimed to address the challenges of mHealth app adoption and sustained use.

**Methods:**

The Rapid Application Development approach was adopted. Stakeholders including decision makers, physicians, nurses, health care administrators, and research engineers were engaged. The process began with requirements gathering to define and finalize the project scope, followed by an iterative process of developing a working prototype, conducting User Acceptance Tests, and improving the prototype before implementation. Co-designing principles were applied to ensure equal collaborative efforts and collective agreement among stakeholders.

**Results:**

*DrCovid+* was developed on Telegram Messenger and hosted on a cloud server. It features a secure patient enrollment and data interface, a multilingual communication channel, and both automatic and personalized push messaging. A back-end dashboard was also developed to collect patients’ vital signs for remote monitoring and continuity of care. To date, 400 patients have been enrolled into the system, amounting to 2822 hospital bed–days saved.

**Conclusions:**

The rapid development and implementation of *DrCovid+* allowed for timely clinical care management for patients with COVID-19. It facilitated early patient hospital discharge and continuity of care while addressing issues relating to data security and labor-, time-, and cost-effectiveness. The use case for *DrCovid+* may be extended to other medical conditions to advance patient care and empowerment within the community, thereby meeting existing and rising population health challenges.

## Introduction

The 2019 novel COVID-19 is an infectious disease resulting from the severe acute respiratory syndrome coronavirus 2 [1]. Declared by the World Health Organization as a pandemic, COVID-19 has both high transmissibility and case fatality rates with more than 250 million reported cases and 5.1 million deaths recorded globally [2]. This has severely burdened the existing health care system by posing additional health care demands; requiring the diversion of resources to diagnose, quarantine, and treat cases; disrupting medical supply chains; and causing a personnel crunch [3].

Current evidence suggests that approximately 80% of patients with COVID-19 experienced mild clinical symptoms suitable for home recovery [4,5]. However, widespread concern surrounding the uncertainty of its pathology and symptoms has resulted in a surge of demand for hospital care [6]. Notwithstanding, at the beginning of the outbreak, hospitals were being used to isolate patients with COVID-19 who were unable to effectively isolate themselves at home, further exerting pressure on global health care resources and systems [5,7]. This called for a sustainable solution to alleviate demands for institutional care for COVID-19 through early inpatient discharge and home isolation while ensuring continuity of care for patients.

Mobile Health (mHealth) is gaining traction as a promising way to advance remote and continuous care during the COVID-19 pandemic [8]. Equipped with various communication features, such as instant text messages and voice and video calls, mHealth allows for telemedicine via real-time individual or group communication [9]. Patients can continue to receive standard clinical care while reducing physical crowding in hospital premises. Likewise, clinicians can provide remote care while minimizing exposure to COVID-19 [10]. Moreover, the use of artificial intelligence in mHealth can potentially alleviate existing clinical loads while ensuring optimal care for patients [11]. For instance, mobile artificial intelligence apps such as *Babylon Health* offers around-the-clock digital tools to help patients monitor their health, obtain health information instantly, and schedule appointments and video calls with clinicians [12]. Chatbots, such as *BotMD*, allow clinicians to have 24/7 access to instant information on drugs, hospital protocols, and clinical tools [13]. Tele-social apps have also been developed to allow for remote patient monitoring and education for patients with COVID-19 [11]. For example, in Singapore, *Doctor Covid,* a chatbot developed on Telegram Messenger, was developed to cater to the country’s multiethnic culture through multilingual capabilities. It can also broadcast critical information and reminders to patients and provide clinical and psychosocial surveillance [14]. This serves to facilitate timely and effective information dissemination and provide remote clinical and social care digitally. Taken together, mHealth apps offer an unprecedented opportunity for remote monitoring and continuity of care and the potential to alleviate existing pressure on hospitals’ resources exacerbated by the COVID-19 pandemic [15].

Attempts to use technology for pandemic response and continuity of care for patients with COVID-19 have been met with various challenges. First, the development of innovations is both costly and labor-intensive. With the high transmission rate of COVID-19, many health care resources were diverted to impede the further spread of the virus, leaving little to invest in innovative solutions to support remote care [16]. Second, the use of innovations is laden with concerns relating to data security and privacy, thus often requiring a state-of-art secure platform to preserve the integrity of the highly protected patient information and may not be feasible in resource-limited countries [17-19]. Third, communication-related barriers such as poor language and digital literacy and the potential breakdown of patient-provider communication may impede the uptake of innovations [16,20,21]. Collectively, although it is essential to develop an mHealth app for remote and continuity of care for patients with COVID-19, it is imperative to ensure cost and time efficiency, security, and intuitiveness with sustained engagement and use by the patients.

We aimed to rapidly develop a tele-social app to facilitate the remote monitoring and continuity of care for patients with COVID-19. This paper describes the rapid development process and the app prototype. We will also critically reflect on the development process and the potential expansion of its use case for the wider population.

## Methods

### Study Design

**Figure 1:**
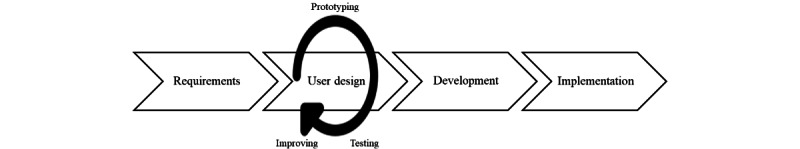
Rapid Application Development process.

The conceptualization of the tele-social app began in September 2021 at the Singapore General Hospital (SGH). It stemmed from the Singapore Ministry of Health’s effort to facilitate the COVID-19 Home Recovery Programme [22], an initiative in Singapore where eligible patients with COVID-19 who have mild to no symptoms were to self-isolate and receive remote care from home. The app was designed to complement the health services in the SGH COVID Virtual Ward (CVW) program, where medically complex patients with COVID-19 who would otherwise isolate in an acute hospital receive remote care and recover at home. To implement the app effectively and efficiently, the Rapid Application Development (RAD) process was adopted (Figure 1). The RAD emphasized an incremental and prototyping approach wherein the continual engagement of stakeholders to obtain user feedback contributes to the generation of further discussion for enhancement; this process continues until a satisfactory system is attained [23]. Collectively, the RAD aims to develop cost-effective yet high-quality innovations in a short duration [23].

The project began with the preparatory phase, where stakeholders engaged in discussions to scope the project. The RAD process then took place with a series of requirements gathering sessions with the relevant stakeholders to define and finalize the project requirements, including the project goals and expectations, timeline, and budget. This information was communicated to research engineers from the Agency for Science, Technology, and Research (A*STAR) for the initial prototype development, followed by an iterative process of User Acceptance Tests and improvement works with key stakeholders before the actual development and implementation.

Co-designing principles were applied throughout the RAD process. Key stakeholders were encouraged to participate actively to understand their needs, opinions, and experiences to ensure the app’s usability and relevance [24] and were respected as equal collaborators, sharing their expertise and having an agreement on the eventual product [25,26].

### Recruitment

Stakeholder engagement began in September 2021. Interdisciplinary and multiprofessional stakeholders—including decision makers, physicians, nurses, and health care administrators operating the SGH CVW and SGH Hospital-at-Home program (a home-hospital program designed to encourage early inpatient discharge and home care)—were invited to join the project team to leverage their knowledge and expertise in home care for patients. Local research engineers from A*STAR, who had prior skills and knowledge in developing the *Doctor Covid* chatbot [14], were also engaged to assist with the designing and development of this mHealth app.

### Data Collection and Analysis

The RAD process involved constant iterations of discussions to capture the requirements, needs, and opinions of key stakeholders. These sessions were conducted via a web-based videoconference platform, Zoom (Zoom Video Communications), in a work group meeting format. Table 1 documents the overview of the discussions, key questions, and outcomes held throughout the RAD phases. All key points were recorded on pen and paper and simultaneously analyzed by 2 research engineers from A*STAR at the end of each work group meeting to facilitate the rapid development. Thematic analysis methodology was adopted wherein similar points were identified and grouped to form themes that informed the features to be included in the mHealth app [27]. This was followed by a series of discussions within the team on the feasibility (ie, whether the feature can be built with existing skills, technology, and time), usability (ie, how user-friendly the features are), and viability (ie, if the feature can be scaled and operated on other similar apps) of the ideas [28].

**Table 1:** Overview of Rapid Application Development (RAD) discussions.

Phase, session, and format		Objectives	Key questions	Outputs
**Preparatory phase**				
	Sessions 1 and 2 (October 6 and 11, 2021); group meetings	Conceptualize requirements for the app Understand the existing use case and explore the enhancement of Doctor Covid to meet the current needs	What are the existing pain points in the workflow processes? What are these pain points that can be addressed by digital tools?	A shared understanding of the basic requirements for the tele-social app A shared understanding of the use case of Doctor Covid The decision to enhance the use case of Doctor Covid for the current project
**Phase 1 (requirements gathering)**				
	Session 3 (October 18, 2021); group meetings	Key stakeholders meeting to deep dive into specific user requirements	What are the specific requirements of users? What are some important considerations in the app (eg, data security and usability)? How and by who is the system being used? What are the patient’s health parameters to track?	Confirmation of digital platform to be leveraged: Telegram Messenger Back-end user dashboard Preliminary user requirements: Automatic push messaging function to communicate medically related information Platform for patients’ daily vital signs uploads User dashboard for health care providers to track patients’ daily vital signs, vaccination status, duration of isolation, discharge date, polymerase chain reaction status, meeting details, and health care provider in charge Security features (eg, secure enrollment and messaging and eligibility requirements)
	Session 4 (October 19, 2021); group meetings	Discuss the patient onboarding process Further discussion on the user requirements	What is the patient flow process? What are some key considerations in remote monitoring?	A shared agreement on the patient workflow (see Figure 1) Additional user requirements: Automatic push reminders to alert patients to upload daily vital signs
	Session 5 (October 21, 2021); group meetings	Presentation of mock-up dashboard by A*STAR^a^ Further discussion on the user requirements	How are patients’ data being tracked and stored? What are some other user requirements?	Additional user’s requirements: Dashboard: enable sorting of patient lists according to demographic variables and a color-coding system to highlight abnormal patients’ vital signs Telegram services: multilingual options Logistics: types of educational videos to be shared with patients
**Phase 2a (developing a working prototype)**				
	Session 6 (October 22, 2021); group meetings	Confirmation of messaging templates, issuing of administrative accounts, and scheduling of UAT^b^ dates	What is your feedback on the translated messaging template?	Messaging templates (eg, onboarding instructions) were confirmed Administrators were issued a testing account Confirmation of UAT dates
**Phase 2b (UAT)**				
	Session 7 (October 29, 2021); group meetings	UAT	What are your feedbacks about the prototype? How do you think this can be further improved?	Feedback for improvements from the UAT: Dashboard: include an overview of the patient list with the total number of patients enrolled and include an export function for patient data Telegram services: messaging reminder timings to be revised
**Phase 2c (improvement works)**				
	Session 8 (November 2, 2021); group meetings	Presentation of improvement works based on feedback received from the UAT	Users to provide any feedbacks	Confirmation of working prototype
**Phase 3 (development and implementation)**				
	Session 9 (November 5, 2021); group meetings	Go live session	N/A^c^	N/A
	Session 10 (November 8, 2021); group meetings	User’s feedback session Discussion of new features	Is there any feedback on the app after initial implementation? What are some new features that are good to have for the next phase?	User’s feedback: Color-coding of patients’ vital signs may not accurately reflect the patient’s existing health status New features: Dashboard: include a read-only function to prevent unnecessary edits made to the dashboard, color-coding system for nonmandatory patient vital signs, and a password option in the user log-in platform Telegram services: include pop-up messages
	Session 11 (November 8, 2021); group meetings	Upgraded go-live version to be released	N/A	N/A

^a^A*STAR: Agency for Science, Technology and Research.

^b^UAT: User Acceptance Test.

^c^N/A: not applicable.

### Ethical Considerations

No ethics review was required for the project as no patients were involved in the design and development of the app. Only health care personnel (ie, physicians, nurses, and health care administrators) and software engineers were involved throughout the process. Moreover, the *DrCovid+* app was incorporated as part of an established home care program (ie, SGH CVW) to provide complementary remote care to patients with COVID-19 and is not part of a human subject research study. The privacy and confidentiality of patient data were ensured, with these data collected only for routine patient care purposes.

## Results

### Overview

Through a systematic and iterative RAD approach, *DrCovid+*, an expansion of the *Doctor Covid* use case [14], was developed. Decision makers, physicians, nurses, health care administrators, and research engineers were involved in the RAD process. Table 2 shows the demographics of the stakeholders involved.

**Table 2:** Participant information.

Phase, session, and format			Sample size, n		Profession		Institution
**Preparatory phase**							
	Sessions 1 and 2; group meetings (via Zoom)	18		Decision makers (n=3) Physicians (n=5) Nurses (n=5) Health care administrators (n=3) Research engineers (n=2)		A*STAR^a^, OST^b^, SHP^c^, and SGH^d^ HaH^e^ team	
**Phase 1 (requirements gathering)**							
	Session 3; group meetings (via Zoom)	5		Nurse (n=1) Health care administrators (n=2) Research engineers (n=2)		A*STAR, OST, and SGH HaH team	
	Session 4; group meetings (via Zoom)	11		Physician (n=1) Nurses (n=5) Health care administrators (n=3) Research engineers (n=2)		A*STAR, OST, and SGH HaH team	
	Session 5; group meetings (via Zoom)	9		Nurses (n=5) Health care administrators (n=2) Research engineers (n=2)		A*STAR, OST, and SGH HaH team	
**Phase 2a (developing a working prototype)**							
	Session 6; group meetings (via Zoom)	10		Physicians (n=2) Nurses (n=2) Health care administrators (n=4) Research engineers (n=2)		A*STAR, OST, and SGH HaH team	
**Phase 2b (UAT^f^)**							
	Session 7; group meetings (via Zoom)	14		Physicians (n=2) Nurses (n=5) Health care administrators (n=4) Research engineers (n=3)		A*STAR, OST, and SGH HaH team	
**Phase 2c (improvement works)**							
	Session 8; group meetings (via Zoom)	14		Physician (n=1) Nurses (n=5) Health care administrators (n=4) Research engineers (n=4)		A*STAR, OST, and SGH HaH team	
**Phase 3 (development and implementation)**							
	Session 9; group meetings (via Zoom)	17		Physicians (n=5) Nurses (n=6) Health care administrators (n=4) Research engineers (n=2)		A*STAR, OST, and SGH HaH team	
	Session 10; group meetings (via Zoom)	13		Physicians (n=2) Nurses (n=5) Health care administrators (n=4) Research engineers (n=2)		A*STAR, OST, and SGH HaH team	
	Session 11; group meetings (via Zoom)	14		Physicians (n=3) Nurses (n=5) Health care administrators (n=4) Research engineers (n=2)		A*STAR, OST, and SGH HaH team	

^a^A*STAR: Agency for Science, Technology and Research.

^b^OST: Office for Service Transformation.

^c^SHP: SingHealth Polyclinic.

^d^SGH: Singapore General Hospital.

^e^HaH: Hospital-at-Home.

^f^UAT: User Acceptance Test.

### Phase 1 of the RAD Process: Requirements Gathering

The project scope, key requirements and challenges were gathered in phase 1.

**Figure 2:**
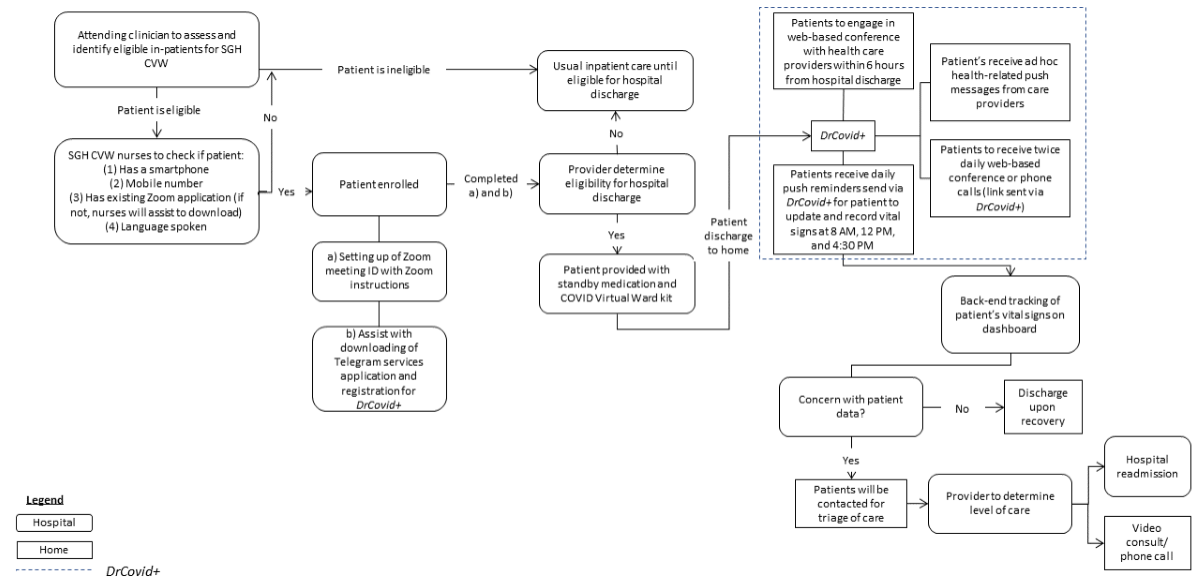
Overview of the Singapore General Hospital COVID Virtual Ward (SGH CVW) workflow.

Given the widespread transmission and the need for close medical attention for patients with COVID-19, *DrCovid+* had to be developed and implemented efficiently and effectively. To achieve this, collaborative decisions were made to develop *DrCovid+* through an expansion of the *Doctor Covid* use case and to incorporate *DrCovid+* into the established work processes of the SGH CVW (Figure 2).

Three priorities were highlighted in phase 1. First, *DrCovid+* would have to be translated into a functional and acceptable prototype, suitable for older adults who were expected to form the majority of the patients in SGH CVW. Second, *DrCovid+* should serve as a remote monitoring app where patients would perform self-reporting of daily vital signs through a web link. Adherence to self-reporting was hence essential and had to be achieved without increasing the existing workload of health care providers. Third, health care providers should be informed at a glance of patients’ latest health status and be able to provide timely follow-up actions where necessary. This resulted in a prototype featuring a multilingual secure communication platform for the remote monitoring and timely dissemination of clinical and social interventions for patients with COVID-19.

### Phase 2 of the RAD process: User Design

#### Overview

*DrCovid+* consists of 2 broad components: an interactive channel where information exchange can occur between the patient and the health care provider via a tele-social app, Telegram Messenger; and a back-end dashboard where health care providers track patients’ health data and send push messages. The app features a secure enrollment platform, automatic push messaging of health reminders and scheduling of web-based conferences, personalized push messaging, and a back-end patient monitoring system.

Telegram Messenger was chosen because of its widespread and ease of use by patients, coupled with its privacy and encryption capabilities and its open-source application programming interface [29]. In addition, Telegram Messenger allows for the flexible customization of bots that facilitates the ease of modification and communication between users and from users to computers. Notwithstanding, Telegram IDs that are unique to users also facilitate the identification of patients without the need to collect patient’s mobile numbers, thereby reducing the risk of information leakage. Moreover, data collected through Telegram Messenger are not integrated with the hospital’s electronic medical records to minimize the risk of data linkages.

The app and back-end servers are hosted on a commercially available cloud server, Amazon Web Services. As the cloud server is readily available, it can be deployed efficiently, and the content and storage can be scaled easily to meet the actual demands without the need to invest in physical infrastructure, which can be costly and time intensive. In addition, security measures are improved with the data security services provided by the commercial cloud vendor (eg, Web application firewire). Notwithstanding, data can be backed up and recovered effectively and efficiently on a cloud storage compared to on-premises storage [29].

#### Enrollment

**Figure 3:**
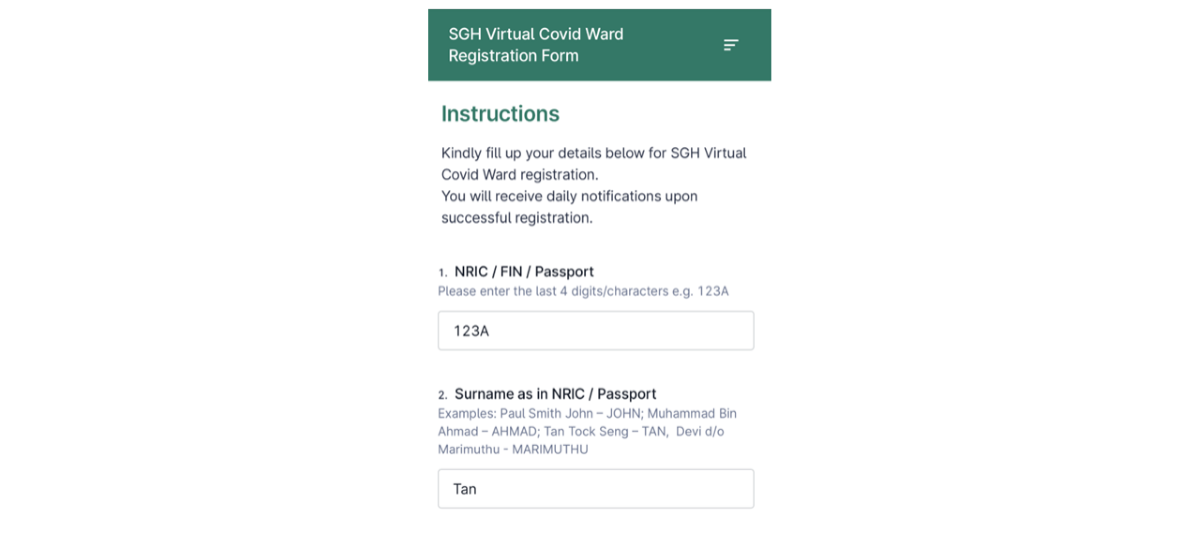
Registration for *DrCovid*+ via FormSG. FIN: Foreign Identification Number; NRIC: National Registration Identity Card; SGH: Singapore General Hospital.

**Figure 4:**
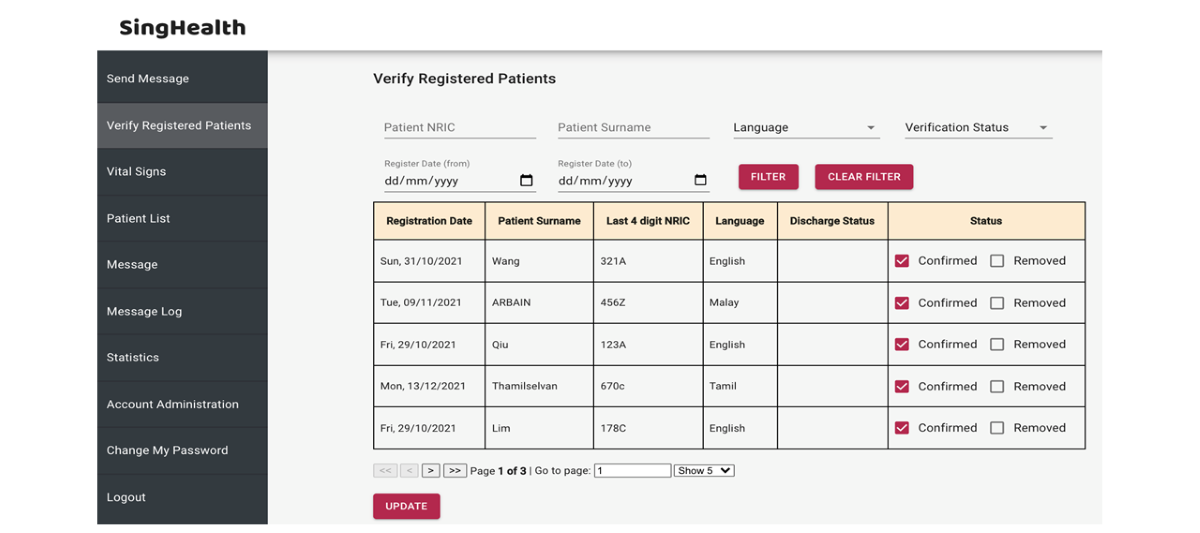
Dashboard for verification and confirmation of enrollment. NRIC: National Registration Identity Card.

To overcome data security issues, eligible patients enrolled in the project are required to register for *DrCovid+* via FormSG, a Singapore government–led web-based data collection form for public officers’ use to preserve the integrity of all patient data [30]. Patients are only required to register for enrollment with the last 4 characters of their National Registration Identity Card number and their last name (Figure 3) for data security purposes. Upon registration, nurses are required to verify and confirm the patient’s enrollment via a corresponding dashboard (Figure 4).

**Figure 5:**
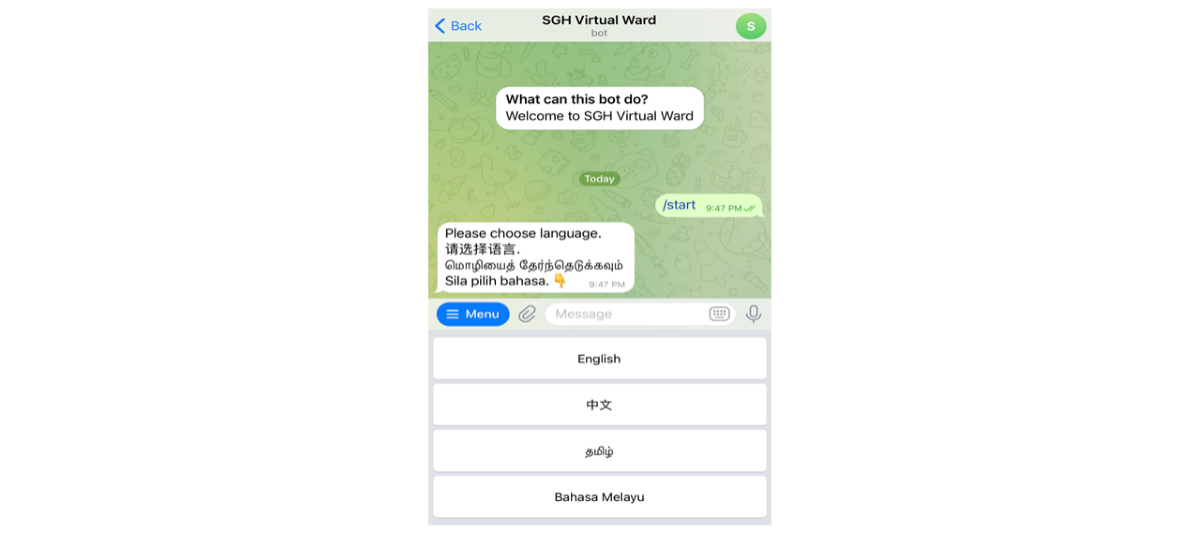
Welcome message on *DrCovid*+. SGH: Singapore General Hospital.

Patients who successfully registered for *DrCovid+* will receive a welcome message through the Telegram app with an automatic prompt to select their preferred communication language. This caters to the multiethnic population as health care providers can tailor messages in a language best understood by the patients (Figure 5).

#### Telegram Services App Interface—Automatic Push Messaging

**Figure 6:**
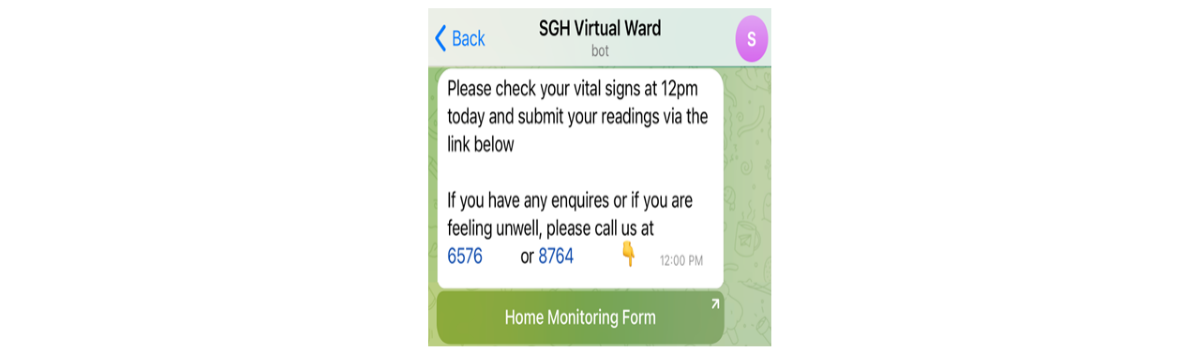
Reminder push message for vital signs submission. SGH: Singapore General Hospital.

**Figure 7:**
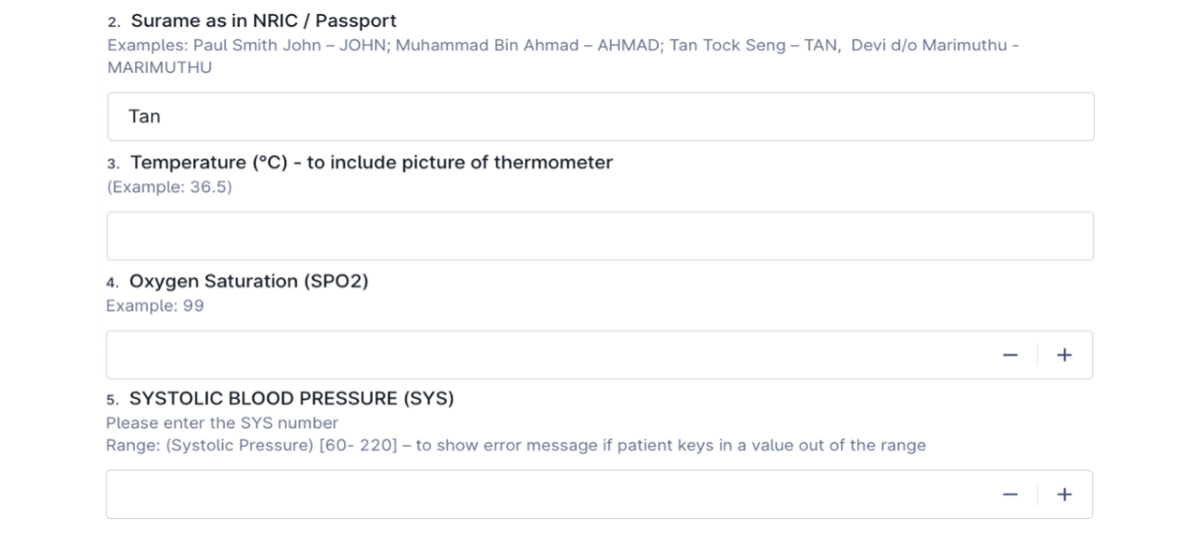
Vital sign updating form on FormSG. NRIC: National Registration Identity Card.

*DrCovid+* serves as a remote monitoring app by prompting patients to measure and report their vital signs daily. An automatic push messaging function on Telegram Messenger is used to send regular reminders at 8 AM, 12 PM, and 4:30 PM to remind patients to monitor and submit the following vital signs through a Home Monitoring Form (Figures 6 and 7): body temperature, heart rate, oxygen level, and blood pressure. The reminders are automatically triggered 30 minutes before the submission time. The care team’s contact information is also included in the reminders, should patients have any queries or concerns. Collectively, this reduced the need for manual prompting from health care providers while ensuring adherence to medical instructions.

#### Back-end Dashboard—Patient Monitoring System

**Figure 8:**
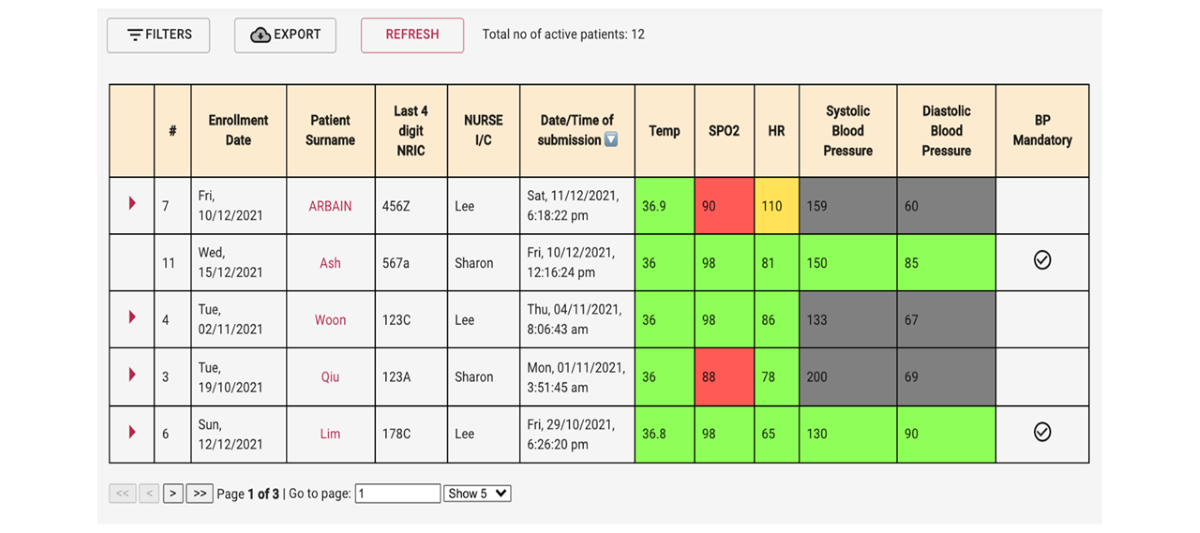
Clinician dashboard for patient's vital signs tracking. BP: blood pressure; HR: heart rate; I/C: in charge; NRIC: National Registration Identity Card; SPO2: oxygen saturation.

Patients’ daily submissions of vital sign readings are sent to a back-end cloud dashboard, monitored by health care providers. The dashboard captures patients’ vital signs and is embedded with logic to highlight abnormal readings in yellow or red, whereas normal and nonmandatory readings are highlighted in green and grey, respectively. This allows for ease of interpretation and timely follow-up actions by the health care providers (Figure 8).

#### Back-end Dashboard—Personalized Push Messaging

**Figure 9:**
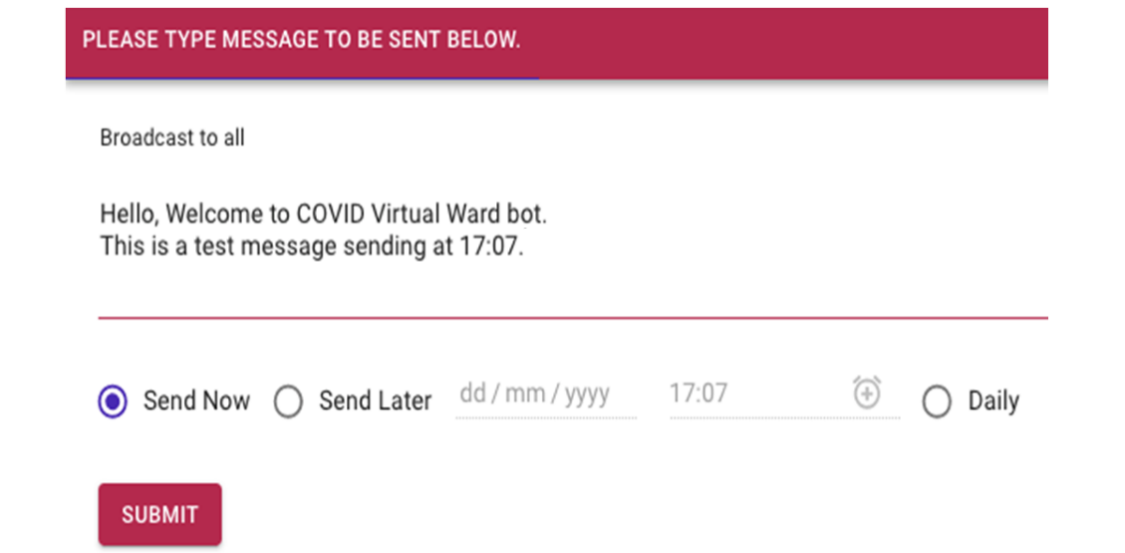
(Test message) Interface for drafting tailored messages.

**Figure 10:**
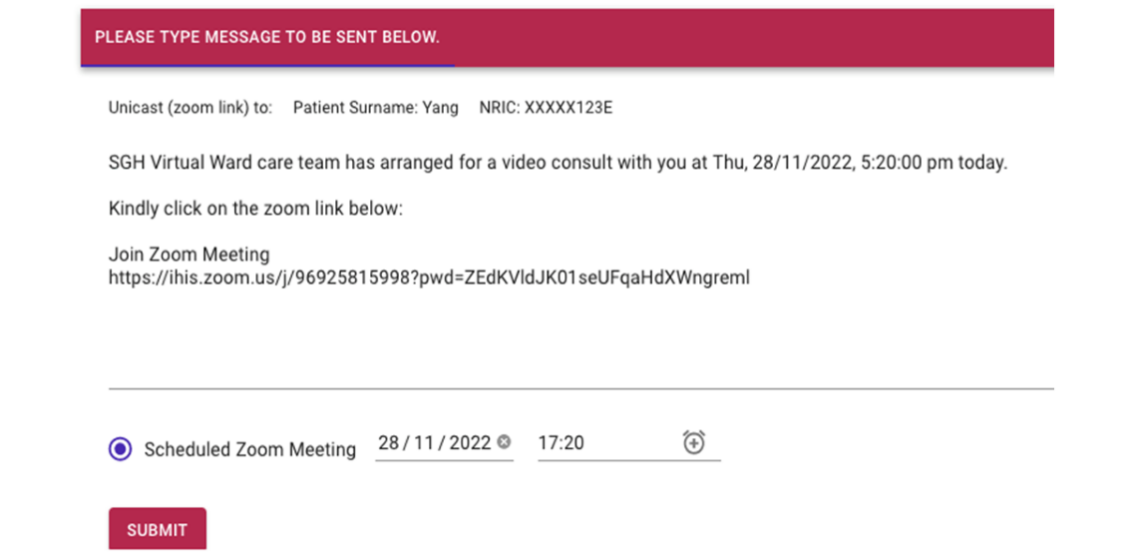
(Test message) Interface for scheduling tailored virtual meetings. NRIC: National Registration Identity Card; SGH: Singapore General Hospital.

**Figure 11:**
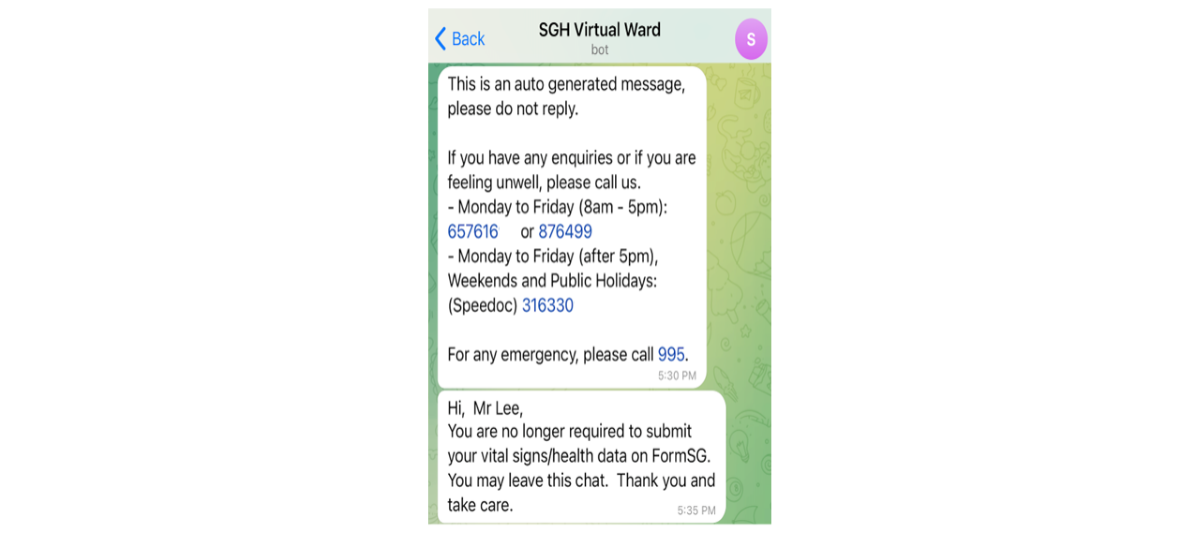
Example of a tailored message on Telegram services. SGH: Singapore General Hospital.

**Figure 12:**
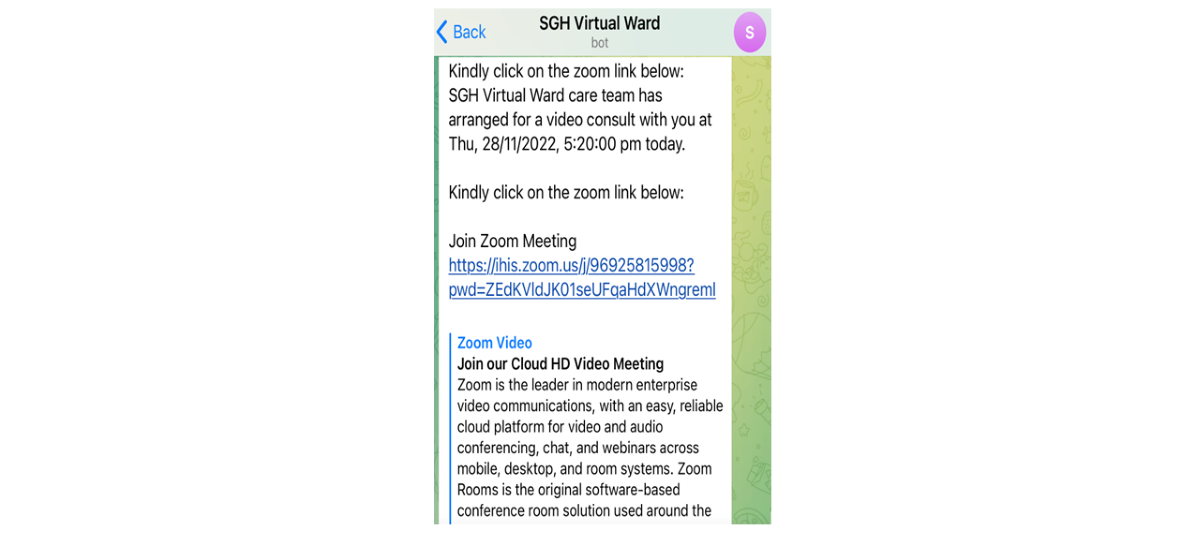
Example of a scheduled ZOOM meeting on Telegram services. SGH: Singapore General Hospital.

As part of the SGH CVW workflow, patients are engaged in 2 to 3 daily web-based consultations with their health care providers. This requires scheduling of web-based consultations and sending the meeting details to the patients. Moreover, health care providers may also need to convey tailored messages or multimedia documents to patients. The back-end dashboard allows health care providers to send these tailored messages to patients at a scheduled time and frequency (Figures 9-12), hence increasing the efficiency of communication with patients and decreasing the workload of health care providers.

### Phase 3 of the RAD process: Development and Implementation

*DrCovid+* has been developed and incorporated into the SGH CVW workflow to complement the remote care services (Figure 2) since October 2021 and has enrolled 400 patients with COVID-19 to date. These are patients who would otherwise be isolated as inpatients and receive care from the hospital premises in the absence of *DrCovid+*. Table 3 illustrates the demographics of the patients. The *DrCovid+* app caters to older adults who are deemed medically stable to receive remote care for their existing medical conditions beyond COVID-19. To date, 94.5% (378/400) of patients enrolled onto the *DrCovid+* app has been discharged, with only 5.5% (22/400) of cases being escalated due to unforeseen medical conditions such as abnormal vital signs readings or the patient having died due to other medical conditions. With *DrCovid+* providing complementary care to the SGH CVW workflow, this has resulted in a shorter total length of stay (LOS), despite an average LOS of 0.64 days more as compared to patients enrolled in the SGH CVW only. The LOS for patients in the *DrCovid+* has contributed to an equivalence of 2822 hospital bed–days saved. Additionally, the remote care capability also reduced the need for routine home visits, contributing to productivity and person-days saved.

Ongoing enhancements and feedback sessions were conducted routinely to ensure the system’s reliability and usability. Although minor technical issues including downtime and crashes were recorded, these issues were escalated and rectified immediately by the research engineering support team.

**Table 3:** Patient characteristics (n=400).

Characteristic		Value
Mean age in years (SD)		51.45 (15.1)
**Gender, n (%)**		
	Male	184 (46)
	Female	216 (54)
**Case escalation^a^ during enrollment in *DrCovid+*, n (%)**		
	Yes	22 (5.5)
	No	378 (94.5)
**Total LOS^b^ (days), n**		
	SGH^c^ CVW^d^ only	3816
	SGH CVW with *DrCovid+*	2822
**Average LOS (days)** **, mean (SD)**		
	SGH CVW only	6.45 (3.69)
	SGH CVW with *DrCovid+*	7.09 (3.53)

^a^Case escalation refers to patients who require inpatient readmission or have died.

^b^LOS: length of stay.

^c^SGH: Singapore General Hospital.

^d^CVW: COVID Virtual Ward.

To further cope with the growing care demands of patients with COVID-19, *DrCovid+* was implemented at Changi General Hospital (CGH), another acute hospital in Singapore since March 2022. CGH was selected as the second site for implementation as it is an academic medical institution located in Eastern Singapore that serves a community of more than 1 million citizens in Singapore. Moreover, the process and requirements for early inpatient discharge and home care for patients with COVID-19 were similar at both CGH and SGH. The implementation process including development, vulnerability assessment test, and security assessment at CGH took only 2 weeks, highlighting the adaptability and flexibility of the app and its capacity for rapid deployment and implementation in a similar setting.

## Discussion

### Principal Findings

As part of the response plan to the COVID-19 crisis in Singapore, this paper documents the RAD process of a tele-social app, *DrCovid+*. Equipped with essential functions such as secure tele-social communication and back-end patient monitoring, the app was codeveloped with key stakeholders to allow for timely remote monitoring and continuity of care for existing patients with COVID-19.

The implementation of *DrCovid+* served to address many of the unprecedented challenges posed by the COVID-19 pandemic. Knowing that COVID-19 spreads predominantly through droplets, there was a need to support early hospital discharge and home recovery as the default care management model for suitable patients to minimize the spread [31] without compromising the quality of care delivery. *DrCovid+* was developed to ensure that patients are adequately cared for remotely, with a corresponding platform to track, monitor, and communicate with patients. Moreover, given the need to divert resources to meet the needs of existing patients and curb the spread of the virus, innovative solutions to manage and care for patients had to be developed efficiently [16]. *DrCovid+* leveraged existing program workflows and processes and the use case of *Doctor Covid* and its engineering team’s expertise to assist with its development and implementation. This expedited the development process, resulting in the completion of *DrCovid+* within a short duration. *DrCovid+* also serves to reduce the personnel required to care for patients. Compared to an inpatient setting where approximately 1 consultant, 5 junior doctors, and 5 nurses are required to care for 20-30 patients, the same number of health care professionals can now care for over 100 patients with such remote monitoring system [32]*.* In addition, this can also contribute to substantial cost savings in the form of bed-days saved, hence improving issues relating to bed crunch. Notwithstanding, with any patient data storage platform, security issues must be addressed to safeguard confidential patient data. Given the need for rapid development and implementation, an elaborate and sophisticated security system may be challenging and inefficient to achieve. Nevertheless, *DrCovid+* has various security measures such as collecting only enrolled patients’ last names and the last 4 characters of their National Registration Identity Card number during registration, restricted access to the back-end dashboard, and manual verification for onboarded patients to minimize any possible security breaches and leakage of patient data. With a large majority of patients enrolled for SGH CVW being older adults, *DrCovid+* is also equipped with multilingual communication functions to prevent communication breakdown and enhance the usability of the app.

mHealth solutions are gaining traction among health care professionals, especially for remote monitoring during the pandemic to manage the increasing caseloads, and this is evident from the existing literature. For instance, *e-CoVig*, a system featuring a mobile app, web/cloud platform, and a device for acquiring patient’s vital signs, was developed, and it allows for real-time monitoring of patients with COVID-19 without the need for direct phone calls and has demonstrated high flexibility, modularity, and accuracy and positive feedbacks from users [33]. Another program, *GetWellLoop*, a remote patient monitoring solution consisting of patient engagement and educational materials, has also been implemented to monitor the symptoms of patients with COVID-19, send daily check-in questions and reminders, and serve as a platform for queries [34]. Similar to the *DrCovid+* app, these programs were designed to be developed rapidly and cost-effectively, serving the needs of the population and the urgent demand for remote care amid the pandemic. However, *DrCovid+* is unique in that it tapped and expanded on existing resources to develop an innovation that serves existing needs. Moreover, despite the limited time frame for development and implementation, a systematic RAD process was in place to ensure that the final prototype adheres to the needs and requirements of its end users.

### Use Case of DrCovid+

The ubiquitous nature of mobile technology coupled with the increasing acceptance of mHealth has provided new windows of opportunity to augment its use in population health. Shifting from the COVID-19 pandemic to other pressing global public health issues such as the aging population, where increasing chronic and complex health care needs are pertinent and care needs extend beyond the hospital walls [35], a concerted effort has to be invested to empower patients and increase access to resources within the community to ensure a sustainable health care system [36]. Tele-social apps such as *DrCovid+* can be a promising innovation to advance patient care and empowerment [37]. Designed to ensure modularity and flexibility, the app can be tailored to suit various use cases, such as sending text reminders for vaccination or medication intake, providing health education materials, and teleconsultations [38-40]. This also addresses the concern on sustained engagement given that *DrCovid+* engages the patient on multiple fronts with different use cases. The adaptation of the use case for *DrCovid+* can potentially minimize unnecessary waiting time and travel expenses for clinic visits and contribute to the reduction in hospital-acquired infection [41,42], making it a promising way forward.

### Strengths and Limitations

The development and implementation of *DrCovid*+ had to be rapid to cope with the increasing COVID-19 caseloads. Despite this constraint, the process of developing the app used systematic RAD processes and co-designing principles such as continual engagement and taking the stance of equal collaboration to ensure the relevance and adoption of the eventual product. Regular meetings were also conducted to gather feedback and suggestions on the grounds for further modifications of the app. The current project, however, requires an evaluation of the cost-effectiveness and overall efficacy as more patients are enrolled. It will also be appropriate to assess time savings for health care providers and the difference in the characteristics of patients who onboarded the *DrCovid+* app compared to controls.

### Conclusions

The impetus to provide remote care and support to the increasing home recovery of COVID-19 cases and the urgency of the COVID-19 situation prompted the need to leverage mHealth technologies and rapid deployment. However, this was met with various challenges including cost-, labor-, and time-intensive requirements in developing an app; data security issues; and poor adoption rates due to communication-related barriers. The *DrCovid+* app was developed to address the pressing demands for remote patient management and support the existing cost and technological bottlenecks. The rapid, cost-effective, yet strategical development of a tele-social app efficiently reaches out to a large number of patients while ensuring timely care provision, and this use case can also be scaled and expanded to meet other population health needs to tackle rising health care challenges.

## Abbreviations

A*STAR: Agency for Science, Technology and Research
CGH: Changi General Hospital
CVW: COVID Virtual Ward
LOS: length of stay
mHealth: mobile health
RAD: Rapid Application Development
SGH: Singapore General Hospital
